# Human amniotic fluid stem cells have a potential to recover ovarian function in mice with chemotherapy-induced sterility

**DOI:** 10.1186/1471-213X-13-34

**Published:** 2013-09-04

**Authors:** Dongmei Lai, Fangyuan Wang, Yifei Chen, Li Wang, Yanlin Wang, Weiwei Cheng

**Affiliations:** 1 The International Peace Maternity and Child Health Hospital, School of medicine, Shanghai Jiaotong University, Shanghai 200030, China

**Keywords:** Human amniotic fluid stem cells, Germ cell, Folliculogenesis, Premature ovarian failure, Chemotherapy

## Abstract

**Background:**

Human amniotic fluid cells (hAFCs) may differentiate into multiple cell lineages and thus have a great potential to become a donor cell source for regenerative medicine. The ability of hAFCs to differentiate into germ cell and oocyte-like cells has been previously documented. Herein we report the potential use of hAFCs to help restore follicles in clinical condition involving premature ovarian failure.

**Results:**

Human amniotic fluid was obtained via amniocentesis, yielding a subpopulation of cloned hAFCs that was able to form embryoid bodies (EBs) and differentiate into three embryonic germ layers. Moreover, culture of EBs in medium containing human follicular fluid (HFF) or a germ cell maturation factor cocktail (FAC), expressed germ cells markers such as *BLIMP1*, *STELLA*, *DAZL*, *VASA*, *STRA8*, *SCP3*, *SCP1*, and *GDF9*. Furthermore, one cell line was grown from clone cells transfected with lentivirus-GFP and displaying morphological characteristics of mesenchymal cells, had the ability to restore ovarian morphology following cell injection into the ovaries of mice sterilized by intraperitoneal injection of cyclophosphamide and busulphan. Restored ovaries displayed many follicle-enclosed oocytes at all stages of development, but no oocytes or follicles were observed in sterilized mice whose ovaries had been injected with medium only (control). Notably, identification of GFP-labeled cells and immunostaining with anti–human antigen-specific antibodies demonstrated that grafted hAFCs survived and differentiated into granulosa cells which directed oocyte maturation. Furthermore, labeling of ovarian tissue for anti-Müllerian hormone expression, a functional marker of folliculogenesis, was strong in hAFCs-transplanted ovaries but inexistent in negative controls.

**Conclusion:**

These findings highlight the possibility of using human amniotic fluid-derived stem cells in regenerative medicine, in particular in the area of reproductive health.

## Background

Premature ovarian failure (POF) is a defect characterized by the premature depletion of ovarian follicles. Approximately 1% and 0.1% of women under 40 and 30 years of age, respectively, experience POF. While at present about 25% of all forms of POF can be classified as iatrogenic and related to cancer treatment, more than 50% of the cases remain idiopathic [1,2]. Most women with POF are infertile and suffer unexpected early-onset menopausal symptoms.

Stem cells constitute a unique population possessing self-renewal potential and the ability to differentiate into a specific tissue cell line under defined incubation conditions [3]. Therefore stem cell therapy may potentially provide an avenue to treat women with POF, reproductive aging and/or poor oocyte quality. In this regard embryonic stem (ES) cells have been proposed as a useful model for studying germ cell development. For instance, human ES cells spontaneously expressed germ cell markers during their differentiation into embryoid bodies (EBs) [4]. Moreover, murine and human ES cells differentiated into germ-line cells, underwent meiosis and produced sperm or oocytes, respectively [5,6]. Regarding the regulation of germ cell development, it was found that *DAZL* (deleted in azoospermia-like, DAZL) gene expression played a primary role in the differentiation of ES cells into primordial germ cells [7,8]. Nonetheless, while *Dazl* expression was required for deriving germ cells from murine ES cells in vitro, this only supported progression through the early stages of meiosis. Thus completion of meiosis required mixing ES cells with minced ovarian tissue and grafting under the kidney capsule of ovariectomized recipient mice to obtain oocytes, albeit at a very low efficiency [9]. Considerable work remains to further define the requirements for in vitro differentiation of ES cells into mature gametes so that these techniques can be clinically applied in regenerative reproductive medicine protocols.

In addition, given the difficulties in growing embryos to obtain human embryonic stem cells, amniotic fluid may be regarded as an alternate source of pluripotent stem cells. Human amniotic fluid contains multiple fetus-derived cell types that possess self-renewal and pluripotency properties. Hence, human amniotic fluid stem cells (AFSCs) have a great potential to become a donor cell source of choice for regenerative medicine [10]. Moreover, human AFSCs display several advantages over ES cells in regards to pluripotency and proliferation rate. For instance, human AFSCs grew extensively in culture and were induced to differentiate into cell types representing different germ layers, that is, into osteogenic, chondrogenic, adipogenic, renal, hematopoietic or neurogenic cell lineages [11]. Furthermore, hAFCs expressed *DAZL*[12], suggesting potential ability to differentiate into germ cell lineage cells. Recently, Cheng et al. reported that human amniotic fluid stem cells cultured in medium containing 5% porcine follicular fluid can differentiate into oocyte-like cells [13].

In this study, we characterized the expression of stem and germ cell markers in undifferentiated hAFCs and observed that germ cell marker expression could be induced in vitro in hAFCs-derived embryoid bodies (EB). Then, to test the functional ability of such cells in vivo, GFP-transfected hAFCs were transplanted into the ovaries of chemically-sterilized mice. Altogether, we highlight a new possibility for using human amniotic fluid cells in regenerative medicine in the area of reproductive health.

## Methods

### Amniotic fluid, follicular fluid and oocyte sample collection

All amniotic fluid cell samples were obtained via amniocentesis performed after the 18th week of pregnancy for routine prenatal diagnosis. The indications were advanced maternal age, a familial or personal history of birth defects, or any foreseen risk of the fetus carrying a chromosomal anomaly or inherited condition. The cytogenetic analyses revealed normal karyotypes for all donors. The mean maternal age was 36 years, with a range of 33–38 years of age, and the mean gestational age was 19 weeks, with a range of 16–22 weeks. Samples of follicular fluid (5 ml) were separately collected from three women (range 29–40 years of age) who were undergoing oocyte pick up for in vitro fertilization and embryo transfer (IVF-ET) to overcome male factor infertility. Ten GV oocytes with fertilization failure were collected from four women (range 32–40 years of age) who were undergoing IVF-ET. This study was carried out with the approval of the Ethics Committee of the International Peace Maternity and Child Health Hospital, Shanghai Jiaotong University, Shanghai, China, and informed consents were obtained from all donors.

### Preparation of follicular fluid

Three samples of follicular fluid (5 ml each) were mixed, pooled and centrifuged at 4000 g for 5 min. The supernatant was filtered (0.22 μm pore size) to eliminate contaminating cells potentially present in follicular fluid, aliquoted and stored at −20°C until use. All experiments were performed with the same batch.

### Human amniotic fluid cell culture and differentiation

Amniotic fluid samples were centrifuged individually at 4000 × g for 5 minutes at room temperature and the supernatant was discarded. Cellular components were grown in DMEM/F12 medium (Gibco, Grand Island, NY, USA) containing 15% ES-FBS (Gibco, Grand Island, NY, USA), 1% glutamine and 1% penicillin/streptomycin (Gibco, Grand Island, NY, USA), supplemented with bFGF (4 ng/ml, Invitrogen, Carlsbad, CA, USA) at 37°C in a 5% CO_2_ atmosphere. After expansion to confluence (5–7 d), AFSCs formed clones. Then, some clones were used to prepare a single cell suspension by gentle trypsinization. In addition, some clones were picked up and cultured under standard EB media supplemented with 5% human follicular fluid for 7–14 days to promote EB formation. Then, EBs were maintained in differentiation medium which contain germ cell maturation factor cocktail (FAC) [9] for 7–14 days by replacing half the medium every 3–4 days. The FAC medium consisted of standard EB media supplemented with a germ cell factor cocktail containing: human SCF 100 ng/ml, SDF1 20 ng/ml, bFGF 20 ng/ml, BMP4 50 ng/ml (all R&D Systems, Minneapolis, Minnesota, USA), N-acetylcysteine 1 mg/ml, forskolin 5 mM, retinoic acid 1 mM (all Sigma, St. Louis, Missouri, USA) and CYP26 inhibitor R115866 1 mM (Johnson & Johnson, Brunswick, New Jersey, USA).

### RNA extraction and real-time qPCR analysis

Total RNA extraction from samples was performed using the RNeasy Mini Kit (Qiagen, Chataworth, CA, USA). Five hundred ng of total RNA from each sample was used in reverse transcription (RT) using the iScript cDNA synthesis kit (Bio-Rad, Hercules, CA, USA). Real-time RT-qPCR was carried out on cDNA using IQ SYBR Green (Bio-Rad, Hercules, CA, USA) on the Mastercycler ep realplex (Germany). All reactions were performed in a 25-μl volume according to the kit. Primer sequences are listed in Additional file 1: Table S1. Cycling conditions were as follows: 1) for *ZPA*, *ZPC*, *GDF9*, *STELLA*, *STRA8*, and *DAZL* 94°C for 2 min, then 94°C for 30 sec, 60°C for 30 sec, 72°C for 45 sec, 28 cycles, then 72°C for 10 min; for *OCT4, CD133, CD117, HLA-DR, BLIMP1, VAZA, SCP1, SCP3, 18 s RNA*, 94°C for 2 min, then 94°C for 30 sec, followed by 28 cycles at 53°C for 30 sec and 72°C for 45 sec, finalizing at 72°C for 10 min; and, 2) for NANOG and cMOS, 94°C for 2 min, then 94°C for 30 sec, followed by 28 cycles at 60°C for 30 sec and 72°C for 45 sec, and finalizing at 72°C for 10 min. The amplification efficiency of different genes was determined relative to 18sRNA (^∆^C t = Ctgene-Ct18sRNA). The quantity of mRNA in each sample was calculated by the comparative (^∆∆^Ct = ^∆^Ctgene-^∆^Ctcontrol) value method. The fold change in gene expression relative to the control was calculated by 2-^∆∆^Ct [14]. Data were obtained by averaging the results from three independent experiments.

### Flow cytometry

The expression of OCT4 and CD117 was evaluated on both single cell suspensions obtained from hAFCs as well as on cells from EBs. A total of 1 × 10^6^ Cells were suspended in 2% BSA/PBS and labeled with the corresponding antibodies, as follows: 1) mouse anti-human OCT3/4 conjugated with Alexa Fluor 488 (eBioscience, San Diego, CA, USA) and 2) mouse anti-human PE-labeled CD117 (eBioscience, San Diego, CA, USA). Identification of Oct4+ and CD117+ cells was performed using a FC500 flow cytometer (Beckman Coulter, Miami, FL, USA) and analyzed by Beckman Coulter CXP software.

### Animals

Forty six-week-old C57BL/6 females were prepared as sterilized recipients by intraperitoneal injection of busulfan (30 mg/kg; resuspended in DMSO) and cyclophosphamide (120 mg/kg resuspended in DMSO) once and were observed for 1 week. Five age-matched females injected with DMSO only were used as non-sterilized controls. All animal procedures were approved by the Institutional Animal Care and Use Committee of Shanghai, and were performed in accordance with the National Research Council Guide for Care and Use of Laboratory Animals.

### Transfection and transplantation of human amniotic fluid cells

Once hAFCs had grown to a density of 80–90%, lentivirus vector with enhanced green fluorescent proteinlenti-EGFP (a gift from Tianjin Liu [15]) was added to cultured cells and incubated at 37°C in a 5% CO_2_ atmosphere for 24 h. Titration of concentrated supernatants was performed by serial dilutions of vector stocks on 1 × 10^5^ Hela cells followed by fluorescence-activated flow cytometry analysis according to the formula: 1 × 10^5^ Hela cell × % EGFP positive cells × 1000/μl virus. Titers of lentiviral vectors were 1 × 10^8^ - 1 × 10^9^ TU/ml. After growing for another 2 days, hAFCs were examined by fluorescence microscopy. The overall cell transfection rate was determined to be greater than 95%. After lentiviral infection for 2 days, hAFCs were washed three times and trypsinized (0.25% trypsin), neutralized in 10% FBS, washed in PBS and resuspended in the culture medium. Recipients were anesthetized by an intraperitoneal injection of pentobarbital sodium (45 mg/kg body weight). Approximately 6 μl of a single-cell suspension, containing 2–5 × 10^3^ cells (n = 30), or 6 μl of culture medium for control (n = 10), was injected into both ovaries of the sterilized recipient. Microinjection of each ovary was performed as previously described according to Zou et al. [16].

### Immunohistochemical analysis

Two months following ovary injection, recipient and control mice were euthanized by cervical dislocation. Ovaries were dissected and fixed in 4% paraformaldehyde (4°C, overnight), dehydrated through a graded series of ethanol, vitrified in xylene, and embedded in paraffin. Six-μm thick sections were fixed for 5 minutes in 10% buffered formalin, after which endogenous peroxidase activity was quenched by incubating the sections in 0.3% hydrogen peroxide in methanol for 30 minutes. Different sections were then incubated in the presence of the following antibodies: mouse anti-human mitochondria (1:300; monoclonal antibody, EMD Millipore, Billerica, Massachusetts, USA); mouse anti-human nuclei (1:100; human nuclei, EMD Millipore, Billerica, Massachusetts, USA); mouse anti-human follicular stimulating hormone receptor (FSHR; 1:100; Abcam, Cambridge, UK); mouse anti-human anti-mullerian hormone (AMH; 1:30 AbD Serotec, Kidlington, Oxford, UK). A peroxidase kit (Vector Laboratories, Burlingame, CA, USA) was used following the manufacturer’s manual. Peroxidase substrate was developed using the DAB (3,39-diaminobenzidine) substrate kit (Vector Laboratories). Slides were counterstained with hematoxylin QS (Vector Laboratories) and were either mounted with low viscosity aqueous mounting medium (Scytek Laboratories, Logan, UT, USA) or dehydrated and mounted with VectaMount Permanent Mounting Medium (Vector Laboratories).

### Immunofluorescence staining

Both individual hAFCs and EBs in suspension were fixed in 4% paraformaldehyde for 15 to 20 min at room temperature, and then washed twice (10 min each) with 1 × PBS. Cells were permeabilized with 0.1% Triton X-100 for 10 min at room temperature, and then washed twice with 1 × PBS. After blocking in 10% skim milk solution for 30 min, cells were incubated with the anti-OCT-4A (rabbit anti-human; 1:200; Santa Cruz, CA, USA), anti-Blimp1 (goat anti-human; 1:200; Santa Cruz, CA, USA), anti-DAZL (goat anti-human; 1:500; Santa Cruz, CA, USA), anti-STELLA (goat anti-human 1:200, Santa Cruz), anti-ZPC (rabbit anti- human; 1:200; Santa Cruz, CA, USA), or anti-SCP3 (rabbit anti-human; 1:800; Abcam, Cambridge, UK) antibodies, for 1 h, at room temperature.

Ovaries from recipient and control mice were fixed with the Tissue-Tek® OCT7™ Compound (Sakura Finetek Middle East, Dubai, United Arab Emirates) and sliced in 5-μm thick sections. Slides were washed twice with PBS and kept in blocking solution for 30 min at room temperature. Slides were then incubated with rabbit polyclonal anti-GFP (1:200; Chemicon, Billerica, Massachusetts, USA) at 4°C, overnight.

Cells or sections were washed three times with 1 × PBS, and probed with FITC-labeled IgG (1:200, Santa Cruz, CA, USA). Fluorescence images were taken using a Leica DMI3000 microscope (Wetzlar, Germany).

### Statistical analysis

Means for relative gene expression were compared by ANOVA using Microsoft Excel software. Statistical significance was set at P < 0.05.

## Results

### The expression level of stem cell and germ cell markers in undifferentiated human amniotic fluid cells

**Figure 1:**
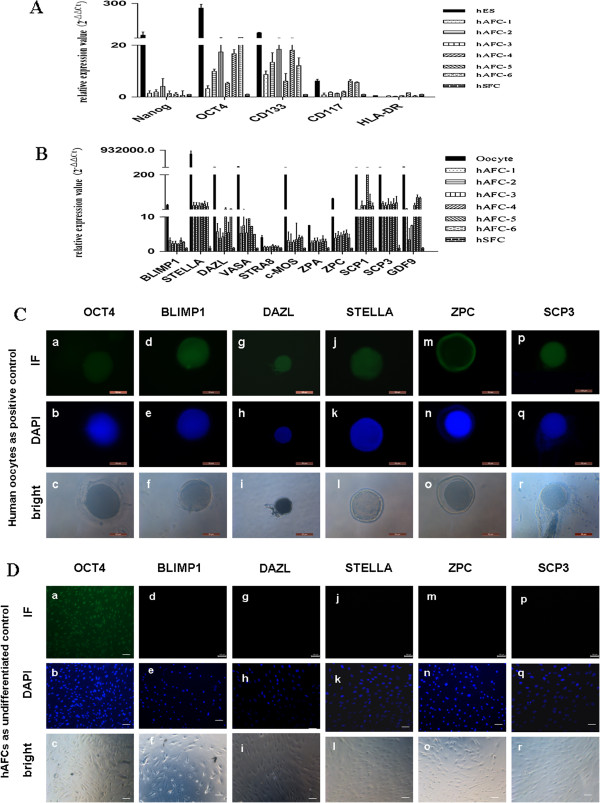
**The expression of stem and germ cell-specific genes in undifferentiated human amniotic fluid cells (hAFCs). (A**,**B)** Quantitative PCR was used to compare stem cell and germ cell specific gene expression in hAFCs obtained from 6 independent samples, human embryonic stem cells (hES) and human GV oocytes. Human skin fibroblast cells (hSFC) were used as negative controls and 18 s RNA was used as an internal housekeeping gene. Results shown represent mean ± standard deviation from three independent experiments. **(C)** Immunofluorescence analysis of germ cell-specific genes in human GV oocytes. Scale bars = 50 μm. **(D)** Immunofluorescence analysis of germ cell-specific genes in undifferentiated hAFCs. While hAFCs expressed OCT4, expression was negative for BLIMP1, DAZL, STELLA, ZPC and SCP3. Scale bars = 50 μm.

To examine germ cell-specific genes in hAFCs, six independent samples obtained via amniocentesis were cultured for one week and a portion of the grown cells were assayed. Real-time qPCR was used to assess gene expression in comparison to human embryonic stem cells (hES; positive controls for stem cells), human oocytes (positive controls for germ cells), and human skin fibroblast cells (hSFCs; negative controls for pluripotency markers). As expected [17,18], all hAFCs samples consistently expressed *OCT4*, albeit at relatively lower levels than hES. Cells originating from amniotic fluid samples also expressed the hematopoietic stem cell marker *CD133*. However, the expression of the major histocompatibility complex (MHC) Class II *HLA-DR* was low in all groups (Figure 1A). These results were consistent with amniotic fluid samples yielding a population of pluripotent cells, given that *OCT4* expression is restricted to pluripotent ES cells [19,20].

Then, we examined the expression of germ cell-specific genes in hAFCs as compared with human oocytes. These genes included: B-lymphocyte-induced maturation protein 1 (*BLIMP1*), *STELLA* and deleted in azoospermia-like *(DAZL)*, which are known to be expressed in PGCs through later stages of germ cell differentiation; VASA which is known to be expressed during gonocyte formation; stimulated by retinoic acid 8 *(STRA8)*, synaptonemal complex protein 1 and 3 (*SCP1, SCP3*), moloney sarcoma oncogene (*c-MOS)*, zona pellucida (*ZP*) gene family (*ZPA, ZPC*) which are expressed during meiosis; and, growth and differentiation factor 9 (*GDF9*), which is an adult oocyte-specific marker [21]. Among the pre-meiotic and meiotic germ cell gene markers, *STELLA* and *SCP1, SCP3* were highly expressed in all six hAFCs samples compared with human skin fibroblast cells, whereas the expression of other same-stage markers (*DAZL, VASA, GDF9*), displayed high variability among samples. Conversely, the expression of *STRA8*, *c-MOS* and *ZPC* was consistently lower in hAFCs samples. Overall, the expression level of the germ cell specific genes was relatively low compared to that in mature oocytes (Figure 1B).

Consistent with the transcriptional profiles, mature oocytes expressed germ cell proteins, including OCT4A, BLIMP1, DAZL, STELLA, ZPC and SCP3 (Figure 1C). However, as evidenced by immunofluorescence, OCT4 protein expression was only detectable in hAFCs (Figure 1D). Altogether, these data suggest that less germ cell gene markers are expressed spontaneously in a subpopulation of hAFCs compared to human mature oocytes.

### Cultured hAFC colonies have the ability to differentiate into three embryonic germ cells

**Figure 2:**
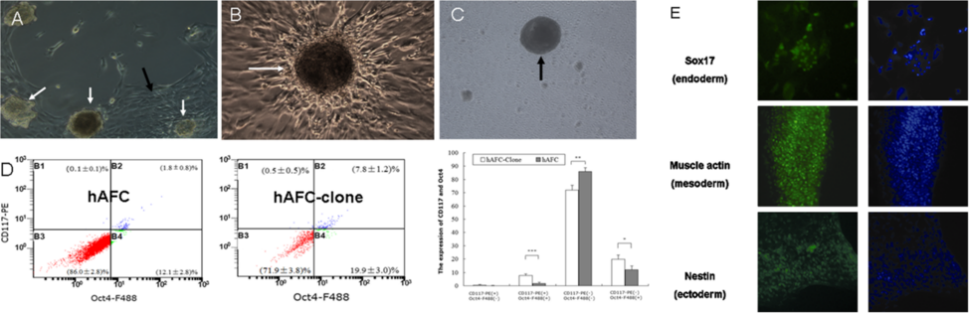
**Phase contrast photomicrographs of human amniotic fluid cultured cells and spontaneous differentiation of clone cells into cells of three embryonic germ layers. (A, B)** hAFCs formed clones (white arrows) and attached cells (black arrows) after 5–7 days in culture. **(C)** Clone cells yielded embryoid bodies (EBs) (black arrow). **(D)** Scatter plots and bar graph for flow cytometry analysis of OCT4 and CD117 expression in attached cells (hAFC) and clones (hAFC-clone). The percentage of Oct4+/CD117+ expressing cells was higher (7.8%) in clone than attached cells (1.81%; ***P < 0.001) (** P < 0.01, * P < 0.05). **(E)** Embryoid bodies spontaneously differentiated into cells stained with Sox17, muscle actin and Nestin respectively. Magnification, 100x **(A)**, 200x **(B**, **C**, **E)**.

Previous work had shown that few cells in human amniotic fluid form colonies under routine cell culture conditions, and while most of the cells in amniotic fluid have the capacity to attach, they do not proliferate or form colonies because of cell cycle arrest, differentiation status, or senescence [22]. Notably, in this study we used a stem cell culture system that supported favorably hAFCs out-growth and that subsequently yielded EB formation. Hence, hAFCs cultured in DMEM/F12 medium supplemented with bFGF for 5–7 days formed clones (Figure 2A-B). Amniotic fluid samples (3–5 ml) yielded inconsistent cell numbers. Hence, after 1 week in culture we obtained 10^4^-10^8^ cells and 10–50 clones per amniotic fluid sample. Clones were digested with accutase (Innovative Cell Technologies, San Diego, CA, USA) each yielding 100–200 cells. Flow cytometry was used to assess OCT4 and CD117 expression in cells stemming from such clones. Whereas over 7% of the clone cells expressed both OCT4 and CD117, only 1.8% of the attached differentiated hAFCs were positive for both transcription factors (P < 0.001, Figure 2C-D).

These clones were also assayed for pluripotency after resuspension in EB media. We observed hAFC-derived EB formation to be accompanied by induction of the differentiation markers Sox17 (endoderm), muscle actin (mesoderm) and Nextin (ectoderm) (Figure 2E), which is agreement with the report of Valli et al. [23]. This demonstrates that clones obtained from hAFCs harbor high differentiation potential.

### Germ cell markers can be induced in cultured hAFCs colones in vitro

In previous studies, the derivation of germ cells relied on the formation of embryoid bodies (EBs) from ES cells. Moreover, differentiation of immature oocytes and sperm from murine and human ES cells in culture has also been reported [4-6]. To determine whether germ cell differentiation could be achieved from hAFCs via EB formation, clones were suspended under EB differentiation medium (Figure 2C). The EB-derived cells were cultured for 1 or 2 weeks in either standard differentiation medium alone or containing a germ cell maturation factor cocktail (FAC) as previously reported [9]. The FAC cocktail comprised antiapoptotic, germ cell specification and meiotic induction factors, including bone morphogenetic protein 4 (BMP4), retinoic acid, CYP26 inhibitor (R115866), stromal cell-derived factor 1 (SDF1), stem cell factor (SCF), and basic fibroblast growth factor (bFGF). We also tested medium supplemented with 5% human follicular fluid (HFF) to investigate its ability to induce the derivation of germ line cells.

**Figure 3:**
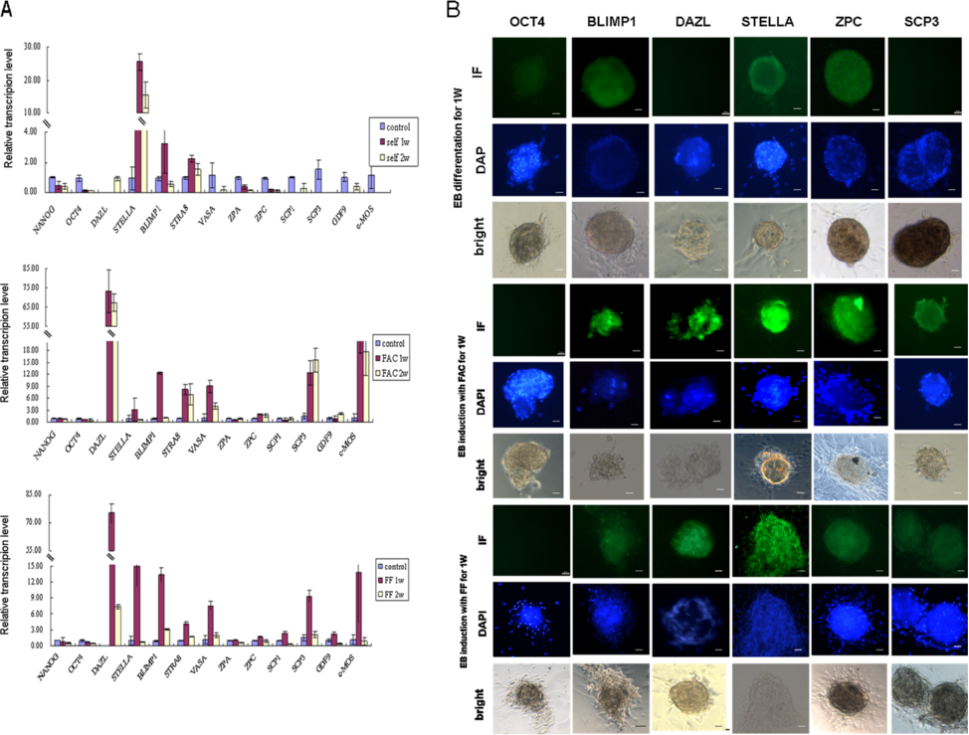
**Gene expression level of germ cell markers in embryoid bodies (EBs) derived from hAFCs and then cultured further for 1 or 2 weeks (1 w, 2 w). (A)** EBs cultured under standard differentiation conditions, cultured in a differentiation conditions with the addition of a germ cell maturation factor cocktail (FAC) or cultured in human follicular fluid (HFF)-containing differentiation medium. EBs not exposed to differentiation conditions were used as negative controls for gene expression; 18 s rRNA was used as an internal housekeeping gene. Data represents means ± SE of 3 independent experiments. **(B)** Immunofluorescence analysis of cellular and subcellular protein expression for germ cell markers in embryoid bodies (EBs) derived from cultured human amniotic fluid cells (hAFCs). EBs were cultured for 1 week in basic differentiation medium, germ cell maturation factor cocktail (FAC)-supplemented differentiation medium or human follicular fluid (HFF)-supplemented differentiation medium Scale bars = 100 μm.

We had noted from the above experiments that undifferentiated hAFCs did not express germ cell-characteristic genes. Herein, qPCR was used to evaluate gene expression in EBs derived from hAFCs and incubated in germ cell differentiation conditions. Under standard differentiation conditions, without FAC or HFF, the expression of *OCT4* and *NANOG* decreased, whereas *BLIMP1*, *STELLA* and *STRA8* increased. Notably, the expression *DAZL*, a gene that has only been shown to function during germ cell formation and/or maintenance, was undetectable both at the mRNA and protein level in EB cells under standard differentiation conditions (Figures 3A-B). Conversely, EBs cultured in differentiation conditions with FAC or HFF, displayed increased expression of all *BLIMP1, STELLA, DAZL, VASA, STRA8, SCP3,* and *c-MOS* but decreased expression of *OCT4* and *NANOG*. In agreement with mRNA expression levels, immunostaining showed increased protein expression for BLIMP1, DAZL, STELLA, ZPC and SCP3 in EBs cultured in FAC or FF-containing medium. As expected, EBs cultured in basic differentiation medium only showed increased immunostaining for BLIMP1, STELLA and ZPC. There was no difference in ZP isoforms mRNA (ZPC, ZPA) or protein (ZPC) expression before and after incubation in germ cell-inducing conditions. The expression of the meiotic factor synaptonemal complex protein-3 (SCP3) was only detected in EBs induced by FAC or HFF (Figures 3A-B). Altogether, these results are consistent with both HFF and FAC inducing germ cell differentiation of hAFC-derived EBs.

### Mesenchymal-like hAFCs can restore morphologically chemotherapy-damaged ovaries

**Figure 4:**
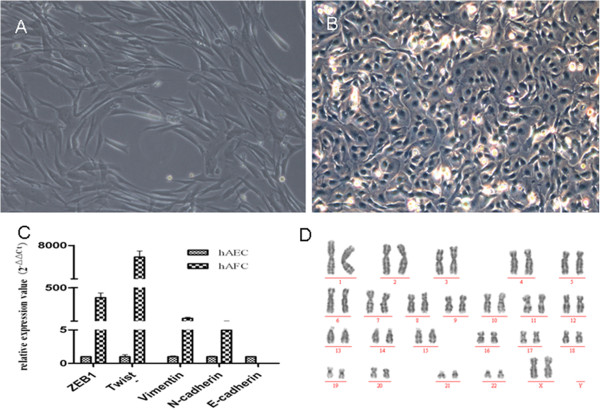
**Characterization of cell type in a human amniotic fluid cell (hAFC) line grown from one sample obtained via amniocentesis. (A)** The hAFCs displayed morphology typical of mesenchymal cells. **(B)** Human amnion epithelial cells (hAEC) grown in culture for comparison. **(C)** Comparison of relative gene expression levels for mesenchymal and epithelial markers relative to 18S RNA; Compared with hAECs, hAFCs cells expressed high levels of mesenchymal markers, such as zinc finger E-box binding homeobox 1 (*ZEB1*), Twist, Vimentin and N-cadherin; conversely, hAFC did not express the epithelial marker E- cadherin. **(D)** Karyotype analysis showed a normal chromosomal complement (46,XX) in hAFCs grown herein. Magnification, 100x **(A)**, 200x **(B)**.

One hAFC line was established from clone cells derived from one sample. Interestingly, the cell morphology mimicked that of mesenchymal cells rather than the typical epithelial morphology of human amnion epithelial cells (hAECs; Figure 4A-B). To further characterize this hAFC line, real-time PCR was used to assay for gene expression of epithelial and mesenchymal cell markers. Compared with hAECs, hAFCs cells derived herein expressed high levels of mesenchymal markers, such as zinc finger E-box binding homeobox 1 (*ZEB1*), twist basic helix-loop-helix transcription factor (Twist), Vimentin and N-cadherin; conversely, hAFC did not express the epithelial marker E-cadherin (Figure 4C). Karyotype analysis revealed a normal chromosomal complement (46, XX; Figure 4D).

**Figure 5:**
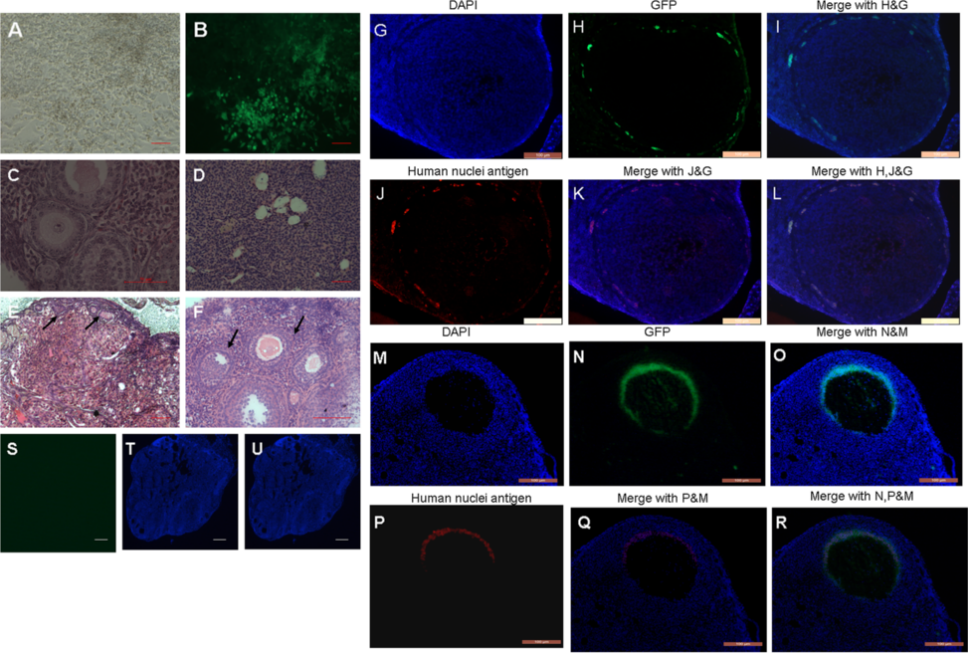
**Transplantation of a line of GFP-transfected human amniotic fluid-derived cells (hAFCs) into chemotherapy-sterilized recipient mice. (A)** hAFC cell line derived from clone cells grown to 85% density. **(B)** GFP-transfected hAFCs. **(C**-**F)** Representative H&E micrographs of ovary sections from: **(C)** non-sterilized normal control mice. **(D)** Sterilized non-transplanted mice after a 2 month-recovery period showing stroma, and atretic primordial or primary follicles. **(E**, **F)** sterilized recipient mice following transplantation of hAFCs; arrows indicate follicles at various stages of maturational development. Sections display primordial **(E)**, as well as primary and large antral follicles **(F)**. **(G**-**R)** Immunofluorescence of ovarian sections from sterilized mice 2 months after transplantation with GFP-transfected hAFCs. **(G**-**I)** GFP staining is observed in ovarian stroma. **(M**-**O)** Follicles containing GFP-positive (green) oocytes. Blue: DAPI immunofluorescence. **(S**-**U)** Culture-medium injected ovaries in control sterilized mice lack GFP signal following a 2-month recovery period. **(G**-**R)** Double-staining with GFP and human nuclei antigen were performed to look for the derivation of GFP positive cells in recipient ovaries 2 months after hAFCs transplantation. **(G**-**L)** Ovarian sections stained with GFP and anti–human nuclei antibody revealing grafted hAFCs in stroma. **(M**-**R)** GFP staining was co-locolized with human nuclei antigen in antral follicles of recipient ovaries after hAFCs transplantation for 2 months. Scale bars = 50 μm **(A**, **B**, **D**, **E**, **S**-**U)**; 100 μm **(G**-**R)**; 200 μm **(C**, **F)**.

Thus these mesenchymal-like cells were used for the transplantation study. To examine the physiological function of hAFCs in vivo, wild-type female mice were sterilized by intraperitoneal pre-treatment with cyclophosphamide and busulphan (n = 40) in order to destroy the existing pre- and post-meiotic germ cell pools [24,25]. Once hAFCs cells had grown to 85% density, they were transfected with lentivirus-GFP (Figure 5A-B). After one week culture, 2–5 × 10^3^ hAFCs were transplanted into both ovaries of the above sterilized recipient mice (n = 30). In addition, control sterilized mice (n =10) were also transplanted with culture medium containing no hAFCs. No transplant-related deaths occurred. Two months following hAFCs transplantation, ovaries were collected and assayed for the presence of oocytes as determined by their morphological appearance and GFP expression. Upon histological evaluation, ovaries injected with hAFCs presented numerous oocytes at all stages of development (Figure 5E-F), similar to the ovaries of non-sterilized mice (n = 5; Figure 5C). Following transplantation, 50% (5/10 mice) of ovaries displayed GFP-positive fluorescence. Interestingly, GFP-positive cells were detected in ovarian tissue nearing oocytes as well as in cells immediately surrounding oocytes (Figure 5G-R). Conversely, two months after sterilization, immature oocytes or follicles were not detected in medium-injected ovaries from control recipients (n = 10, Figure 5D). Furthermore, control ovaries (sterilized but untreated ovaries) comprised stromal and interstitial cells, as well as a few atretic follicles lacking GFP expression (Figure 5S-U), consistent with chemotherapy having destroyed oocytes or ovarian follicles. Altogether, these findings were consistent with hAFCs transplantation restoring folliculogenesis in chemically-damaged murine ovaries.

### Implanted hAFCs infiltrate the chemically-damaged murine ovarian tissue and restore ovarian function

Human-specific antigens, including human nuclei antigen [26] and human follicle - stimulating hormone receptor (FSHR) [27] were further used as tracking markers for transplanted hAFCs. To confirm whether GFP-positive cells in recipient ovaries were indeed derived from grafted hAFCs, we performed double-staining with GFP and human specific nuclei antigen in recipient ovarian sections two months following hAFCs transplantation. Results showed that GFP positive staining did co-localize with human anti–nuclei staining in ovarian stroma (Figure 5G-L) or in antral follicles (Figure 5M-R).

**Figure 6:**
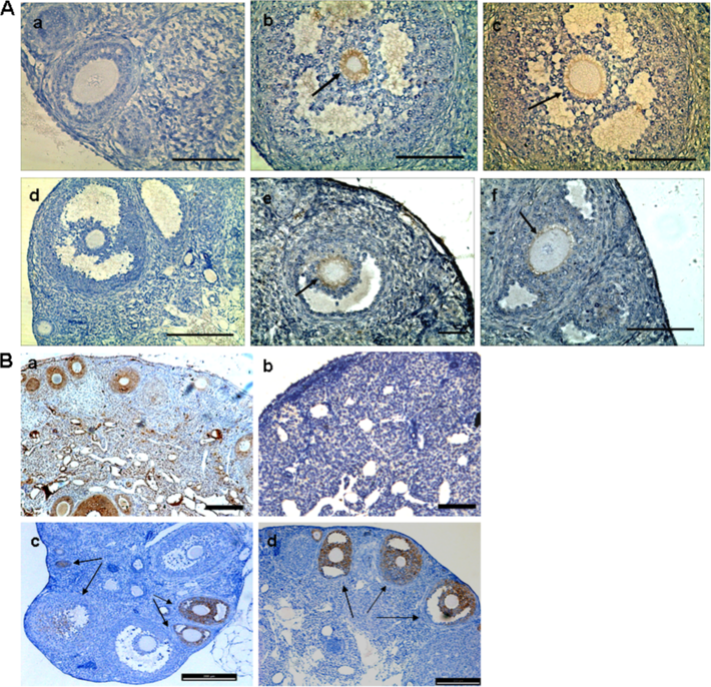
**Grafted hAFCs infiltrate the chemically-damaged murine ovarian tissue and restore ovarian function. (A)** Immunohistochemistry for human-specific antigens detecting grafted cells resulting from transplantation of human amniotic fluid cells into the ovaries of chemically-sterilized mice. **(a)** Sample for human nuclei antigen-negative ovarian section following hAFC transplantation. **(b**, **c)** Human nuclei antigen expression in recipient ovaries 2 months after hAFCs transplantation. **(d)** Sample for human follicle stimulating hormone receptor (FSHR)-negative ovarian section following hAFCs transplantation. (K, L) Human FSHR expression in recipient ovaries 2 months after hAFCs transplantation. Arrows indicate positive expression of human nuclei or human FSHR in the oocyte, while the other granulosa cells served as negative controls. Scale bars = 100 μm. **(B)** Immunohistochemistry for anti-mullerian hormone (AMH) in ovarian sections from mice. **(a)** AMH is expressed in the granulosa cells of primary, preantral and small antral follicles in ovaries from control fertile mice. **(b)** AMH expression disappears in the ovaries of chemically-sterilized mice examined 2 months following hAFC-free medium transplantation. **(c**, **d)** AMH expression reappears in ovaries from chemically sterilized mice when examined at 2 months following hAFC transplantation. Scale bars = 200 μm

To assess the survival and differentiation of the grafted hAFCs, the expression of human nuclei antigen was evaluated by immunohistochemistry. Interestingly, human nuclei expression was detected mainly around the oocytes in antral follicles, similar to the pattern observed for GFP expression (Figures 6Ab-c and 5M-R). These results suggest that hAFCs-derived cells infiltrated the chemically-damaged murine ovarian tissue and differentiated into the somatic but not the germ cell population. Hence, human FSHR, a granulosa cell marker required for normal ovarian development and follicular maturation, was used to further characterize the grafted cells. Interestingly, FSHR could be detected in cells around oocytes, similar to the expression pattern observed with human nuclei (Figure 6Ae-f). Altogether, these results support the notion that a portion of hAFCs integrated into the ovarian niche of sterilized mice and differentiated into granulosa cells.

Anti-Müllerian hormone (AMH) is a protein expressed by granulosa cells that controls the formation of primary follicles by inhibiting excessive follicular recruitment by FSH. Thus AMH levels are strongly correlated with the size of the follicle pool, and can be used as a marker for ovarian aging, responsiveness and pathophysiology [28]. As shown in Figure 6Ba, AMH expression was strong in granulosa cells of preantral and small antral follicles, and gradually diminished in subsequent stages of follicle development in control ovaries from non-sterilized mice. Conversely, AMH was no longer expressed in ovaries from mice sterilized by chemotherapy (Figure 6Bb) but reappeared in ovarian follicles from sterilized mice when evaluated at 2 months following hAFC transplantation (Figure 6Bc-d).

Altogether, the above presented results suggest that hAFCs integrated into the ovaries of infertile mice and participated in oocyte regeneration. Thus ovarian function can be at least partially restored following hAFC transplantation.

## Discussion

**Figure 7:**
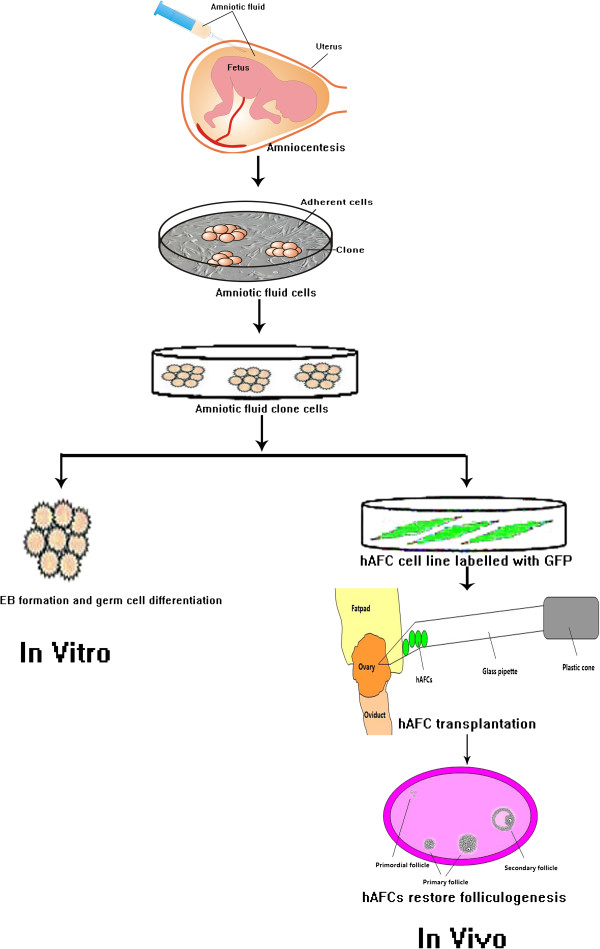
The flow diagram of hAFCs restoring ovarian function.

While ES cells provide a potential avenue for germ cell regeneration in reproductive medicine, their preparation and use still presents many challenges as well as ethical concerns. As an alternative, cells derived from human amniotic fluid (hAFCs), have been shown to exhibit high differentiation potential and high proliferative activity, both characteristics of stemness. Moreover, hAFCs express the stem cell marker OCT4 [9,18,29-32] and can differentiate into cells of all three embryonic tissue layers [9,29-32]. For instance, hAFSCs descending from one single OCT4- and NANOG-positive line were induced to undergo adipogenic, osteogenic and neurogenic differentiation [33]. Similarly, monoclonal AFSCs expressing the markers OCT4 and CD117 (c-kit), could be induced to differentiate into adipogenic, osteogenic, myogenic, endothelial, neurogenic and hepatic cell lineages [11]. Therefore, hAFCs have great potential to become an important source of stem cells for use both in basic research as well as for regenerative medicine purposes. Herein we report the potential of cloned-hAFCs to express germ cell markers in vitro, and the potential of hAFC line derived from cloned- hAFCs to support follicle and oocyte development in vivo (Figure 7).

Firstly, we analyzed hAFC samples for the expression of germ cell markers; in agreement to previous reports [11], we observed a consistently high expression of OCT4 and CD133. However, when we examined additional markers we did not observe expression of genes specific to germ cell differentiation. In this regard, it was reported that hAFCs had the ability to form embryoid bodies (EBs) when cultured under conditions without anti-differentiation factors and without contact to feeder cells. Moreover, the formation of such three-dimensional multicellular aggregates was accompanied by a decrease in stem cell marker expression and by the induction of differentiation into different cell lineages [23]. Hence herein hAFCs kept under a stem cell culture system yielded a high number of clones that when cultured in suspension without anti-differentiation factors, spontaneously formed multicellular EBs and harbored high differentiation potential. Moreover, when we cultured EBs in the presence of germ cell maturation FAC [9] or 5% HFF, we were able to induce expression of markers consistent with germ cell differentiation.

Indeed, both the germ cell maturation FAC as well as HFF appeared to share factors that could potentially induce the differentiation of human amniotic fluid-derived stem cells into germ cell lineage cells. For instance, HFF, which is produced during folliculogenesis, contains factors that are secreted from granulosa and theca cells, as well as oocytes [33,34]. Many growth factors and hormones such as activin A, follistatin, SCF, bFGF, AMH and estrogen have been isolated from follicular fluid [35,36]. In fact, some of these factors, such as SCF and bFGF are also components of the germ cell maturation FAC. The roles of these endocrine–paracrine factors in the regulation of follicular development are well documented [37,38]. Furthermore, previous research had demonstrated that 5% porcine follicular fluid could induce porcine skin-derived stem cells to form germ cells [39]. Our research further supported these notions in that both HFF and FAC-supplemented media efficiently induced hAFCs to express germ cell-specific genes and hence direct them to enter the germ cell path of differentiation.

While several studies have demonstrated that human ES cells can be induced to differentiate into somatic and germ cell lineages, including germ cell precursors or PGCs [40,41], germ cell differentiation has been largely limited to the earliest stages. Therefore, ES cell-derived oocyte maturation ultimately fails in vitro [42-44]. Nicholas et al. transplanted ES cell-derived germ cell co-aggregated with wild type newborn ovarian tissue into the kidney capsule of recipient mice and found that the ovarian niche could direct functional maturation of these germ cells [9]. Similarly, isolated female germline stem cells from neonatal mouse ovaries underwent oogenesis and resulted in offspring when transplanted into ovaries of infertile mice [16]. Hence we hypothesized that hAFC-derived stem cells might differentiate into follicles and hence restore folliculogenesis after transplantation to the appropriate niche, that is, into chemically-damaged ovaries. Thus, we established one hAFC line derived from clone cells of one sample, and used for ovarian transplantation.

In order to follow these cells through development, hAFC line were transfected with lentivirus-GFP prior to transplantation. Indeed, GFP-positive cells integrated into the ovarian niche of infertile mice and this was further supported by the identification of human-specific antigen expression, including human nuclei and FSHR in recipient ovaries. Folliculogenesis depends not only on the circulating levels of the gonadotropins but also on the expression of gonadotropin receptors by follicular cells in the ovary. Because human FSHR, which has been localized to granulosa cells of Graafian follicles [27] was expressed in the ovaries from sterilized mice, this strongly suggested that grafted hAFCs differentiated into granulosa, but not germ cells. Moreover, immunofluorescence detected the expression of human-specific antigens only in the population of follicular supporting cells. Hence, these results strongly support the notion that implanted hAFCs directed follicle formation in the chemically-damaged murine ovary. Notably, increased expression of AMH, a hormone strongly correlated with the size of the follicular pool [28,45,46], was marked after hAFCs transplantation in the ovaries of sterilized mice. Conversely, AMH expression is negative in the control groups, further supporting a role of hAFC transplantation in restoring ovarian function in infertile mice.

Interestingly, not all transplanted ovaries from sterilized mice developed GFP(+) or human antigen-positive cells, but we also identified GFP(−) and human antigen-negative follicular growth. These findings suggested that in these cases transplanted hAFCs may have aided in folliculogenesis recovery via indirect trophism on ovarian tissue. This is consistent with studies spanning other organs where improved functionality was observed not due to cell differentiation but to mere supportive effects on existing cells [47,48].

Worth noting, amniotic fluid-derived cell clones displayed a normal karyotype and no evidence of tumorigenic effect in vivo (Data not shown). In agreement, it was previously reported that hAFCs are genomically stable and harbor neither epigenetic memory nor somatic mutations of already differentiated source cells [11,29-32]. Moreover, compared to ES cells, there is no ethical and religious concerns associated with hES cells which require the destruction of human embryos [49]. In addition, another promising renewable source of pluripotent cells is constituted by induced pluripotent stem cells (iPSCs), which are obtained from reprogrammed somatic cells using defined factors. However, iPSCs can induce a T-cell-dependent immune response in syngeneic recipients [50]. In this regard, work from others revealed that amniotic fluid-derived cells, hold immunosuppressive properties [51] making them even more attractive for their potential clinical applications.

## Conclusions

This study demonstrated hAFCs obtained from early second trimester stages of fetal development may hold the intrinsic ability to express germ cell markers in vitro and restore folliculogenesis in a germ cell-ablated mouse model. The methods outlined above may provide a platform to elucidate the mechanisms of ovarian function restoring. Altogether, our findings have implications for the future use of stem cells in the treatment of POF and aging-related infertility.

## Abbreviations

hAFCs: Human amniotic fluid cells; EBs: Embryoid bodies; HFF: Human follicular fluid; FAC: Germ cell maturation factor cocktail; POF: Premature ovarian failure; IVF-ET: In vitro fertilization and embryo transfer; hSFCs: Human skin fibroblast cells; hAECs: Human amnion epithelial cells; FSHR: Follicle-stimulating hormone receptor; AMH: Anti-Müllerian hormone.

## Competing interests

The authors declare that they have no competing interests.

## Authors’ contributions

DL, YW, WC acquired, analyzed and interpreted the data, and elaborated the manuscript. FW did the cell differentiation in vitro and in vivo. YC did the PCR and immunofluscence. DL participated in the design and interpretation of experiments, and wrote the manuscript. All authors read and approved the final version of the manuscript.

## Supplementary Material

Additional file 1: Table S1 PCR primer sequences. Click here for file
