# Supplementary material for: Human amniotic fluid stem cells have a potential to recover ovarian function in mice with chemotherapy-induced sterility
Source: BMC Dev Biol. 2013 Sep 4;13:34. doi: 10.1186/1471-213X-13-34 (PMC3844331; doi:10.1186/1471-213X-13-34)
Supplement: Additional file 1: Table S1 — PCR primer sequences. [file 1471-213X-13-34-S1.doc]

**Additional file**

Additional file 1: Table S1. PCR primer sequences

| Gene product | Forward(F) and reverse(R) primers(5’ → 3’) | Size(bp) |
| --- | --- | --- |
| *NANOG* | F: GGGCCTGAAGAAAACTATCCATCC  R: TGCTATTCTTCGGCCAGTTGTTTT | 400 |
| *OCT4* | F: GGCCCGAAAGAGAAAGCGAACC  R: ACCCAGCAGCCTCAAAATCCTCTC | 224 |
| *CD133* | F:TGGATGCAGAACTTGACAACGT R:ATACCTGCTACGACAGTCGTGGT | 120 |
| *CD117* | F:CCACACCCTGTTCACTCCTT  R:TTCTGGGAAACTCCCATTTG | 206 |
| *HLA-DR* | F: CAGTTCCTCGGAGTGGAGAG  R: CTCAGCATCTTGCTCTGTGC | 115 |
| *BLIMP1* | F: AAGTGTAACTCCAGCACTGTG  R: CCAAAACGTGTGCCCTTTGGTATG | 290 |
| *STELLA* | F: CTCAAATCTCCTCCGAGACG  R: TTCGATTTCCCTGAGGACTG | 137 |
| *DAZL* | F: AATGACGTGGATGTGCAGAA  R: AACTGTGGTGGAGGAGGATG | 152 |
| *VASA* | F: TTGGGAAGCAGAAATCAACC  R: AAACCACCCATTGTGGATGT | 240 |
| *c-MOS* | F: CGGTGTTCCTGTGGCCATAA  R: AGCAGGCCGTTCACAACATC | 330 |
| *STRA8* | F:TCGTCTCCGCGGCCATCTCC | 153 |
|  | R: TGTCCTTCACGCTGCCCTCG |  |
| *ZPA* | F:GCAGCACTTACCTTGCTTCC | 167 |
|  | R: TCTGGTGGTCACAGCTTCAG |  |
| *ZPC* | F:GCAGGCATGTGACAGAAGAA | 106 |
|  | R:GAGGTGTCAGAAGGCAAAGC |  |
| *SCP1* | F: CAAAAGCCCTTTGCATTGTT | 225 |
|  | R: CTCAAACACGGGCAAGAAAT |  |
| *SCP3* | F: TATGGTGTCCTCCGGAAAAA | 238 |
|  | R: AACTCCAACTCCTTCCAGCA |  |
| *GDF9* | F: GCACAGGTACAACCCTCGAT | 122 |
|  | R: GCACTGAGGAGTCCAGCTTC |  |
| E-cadherin | F: 5-GCCTCCTGAAAAGAGAGTGGAAG-3 | 130 |
|  | R: 5-TGGCAGTGTCTCTCCAAATCCG-3 |  |
| Vimentin | F: 5-AGGCAAAGCAGGAGTCCACTGA-3  R: 5-ATCTGGCGTTCCAGGGACTCAT-3 | 99 |
| Twist | F: 5-CACTGAAAGGAAAGGCATCA -3 | 109 |
|  | R: 5- GGCCAGTTTGATCCCAGTAT -3 |  |
| N-cadherin | F: 5-GACGGTTCGCCATCCAGAC -3 | 66 |
|  | R: 5-TCGATTGGTTTGACCACGG -3 |  |
| *ZEB1* | F: 5-TGCACTGAGTGTGGAAAAGC-3 | 236 |
|  | R: 5-TTGGTGATGCTGAAAGAGACG-3 |  |
| *18s RNA* | F: CGTTGATTAAGTCCCTGCCCTT  R: TCAAGTTCGACCGTCTTCTCAG | 202 |

**Methods**

*Preparation of human skin fibroblast cells (hSFCs)*

Scar skin samples from one patient with past surgical history were obtained with written and informed consent, placed in Hank’s balanced salt solution (HBSS) and cut into ~ 1-mm^2^ pieces using dissecting scissors. Then, the segments were digested in 0.25% trypsin/EDTA at 37 ^o^C, for 45 min. The resulting cell suspensions were seeded in a six-well plate in RPMI 1640 medium (Gibco, Grand Island, NY, USA) supplemented with 10% FBS (PAA Laboratories GmbH, Pasching, Austria), streptomycin (100 U/mL; Gibco, Grand Island, NY, USA), penicillin (100U/Ml; Gibco, Grand Island, NY, USA), and glutamine (0.3mg/Ml Gibco, Grand Island, NY, USA), and incubated at 37 ^o^C in 5% CO_2_ in humidified air. Once hSFCs had grown to a density of 80–90% they were used for PCR assay.

*Preparation of human amnion epithelial cells (hAECs)*

Human placentas were obtained at delivery during uncomplicated caesarean sections with written and informed consent from woman who tested negative for HIV-I, as well as hepatitis B and C. The institutional ethics committee approved the use of human amnion for this project. The amniotic membranes were mechanically peeled from the chorionic portion of the placenta. The membrane was then placed in a 250-mL flask containing RPMI 1640 medium and cut with a razor to yield 0.5–1.0 cm^2^ segments. The segments were digested with 0.25% trypsin/EDTA at 37^o^C for 45 min. The resulting cell suspensions were seeded in a six-well plate in RPMI 1640 medium supplemented with 10% FBS (PAA Laboratories GmbH), streptomycin (100U/mL; Gibco, Grand Island, NY, USA), penicillin (100U/Ml; Gibco, Grand Island, NY, USA), and glutamine (0.3mg/Ml; Gibco, Grand Island, NY, USA), and incubated at 37 ^o^C 5% CO_2_ in humidified air. Once hAECs had reached 80–90% confluence, PCR was performed.

*In Vitro Differentiation of* *Colonies of Human Amniotic Fluid*

Colonies of human amniotic fluid were removed from the standard culture system and cultured under feeder-free conditions under standard EB media. After 5–14 days spontaneous differentiation was observed, and differentiated cells were Cells were fixed in 4% paraformaldehyde in PBS (Sigma) for 30 minutes, and then permeabilized for an additional 10 minutes with 0.1% Triton X (Sigma). The blocking step was 30 minutes with 2% FCS in PBS. Differentiated cells were incubated with antibody against Nestin (1:200; Santa Cruz), Sox-17 (1:200, Santa Cruz), or muscle actin (1:200, Santa Cruz) for an additional 2 hours. Each antibody was detected by using the corresponding secondary antibodies conjugated to FITC (Santa Cruz). The nuclei of cells were stained with DAPI (4', 6-diamidino-2-phenylindole) for 5 minutes. Fluorescence images were taken using a Leica DMI3000 microscope (Wetzlar, Germany).

*Karyotype analysis*

Chromosome analysis of the hAFCs cell line was performed using the G-band methods.
